# Taxifolin: A Potential Therapeutic Agent for Cerebral Amyloid Angiopathy

**DOI:** 10.3389/fphar.2021.643357

**Published:** 2021-02-12

**Authors:** Satoshi Saito, Masashi Tanaka, Noriko Satoh-Asahara, Roxana Octavia Carare, Masafumi Ihara

**Affiliations:** ^1^ Faculty of Medicine, University of Southampton, Southampton, United Kingdom; ^2^ Department of Neurology, National Cerebral and Cardiovascular Center, Suita, Japan; ^3^ Department of Physical Therapy, Health Science University, Fujikawaguchiko, Japan; ^4^ Department of Endocrinology, Metabolism, and Hypertension Research, Clinical Research Institute, National Hospital Organization Kyoto Medical Center, Kyoto, Japan

**Keywords:** IPAD, clinical trial, treatment, Alzheimer’s disease, cerebral amyloid angiopathy, Taxifolin

## Abstract

Cerebral amyloid angiopathy (CAA) is characterized by the accumulation of β-amyloid (Aβ) in the walls of cerebral vessels, leading to complications such as intracerebral hemorrhage, convexity subarachnoid hemorrhage and cerebral microinfarcts. Patients with CAA-related intracerebral hemorrhage are more likely to develop dementia and strokes. Several pathological investigations have demonstrated that more than 90% of Alzheimer’s disease patients have concomitant CAA, suggesting common pathogenic mechanisms. Potential causes of CAA include impaired Aβ clearance from the brain through the intramural periarterial drainage (IPAD) system. Conversely, CAA causes restriction of IPAD, limiting clearance. Early intervention in CAA could thus prevent Alzheimer’s disease progression. Growing evidence has suggested Taxifolin (dihydroquercetin) could be used as an effective therapy for CAA. Taxifolin is a plant flavonoid, widely available as a health supplement product, which has been demonstrated to exhibit anti-oxidative and anti-inflammatory effects, and provide protection against advanced glycation end products and mitochondrial damage. It has also been shown to facilitate disassembly, prevent oligomer formation and increase clearance of Aβ in a mouse model of CAA. Disturbed cerebrovascular reactivity and spatial reference memory impairment in CAA are completely prevented by Taxifolin treatment. These results highlight the need for clinical trials on the efficacy and safety of Taxifolin in patients with CAA

## Introduction

Cerebral amyloid angiopathy (CAA) refers to the abnormal accumulation of amyloid proteins in the walls of cerebral vasculature (Love et al., 2014; Saito et al., 2020b). Seven amyloid proteins have so far been reported in CAA including β-amyloid (Aβ), cystatin C, transthyretin, gelsolin, prion protein, ABri/ADan and immunoglobulin light chain amyloid (Yamada, 2015). The most common form is Aβ-type CAA, which is present in over 90% cases of sporadic, non-familial age-related Alzheimer’s disease (AD) (Saito and Ihara, 2016). The shared role of Aβ deposition in AD and CAA implies interaction between neurodegenerative and cerebrovascular processes (Saito et al., 2015). In this review, we discuss the pathophysiological basis of such interactions and how Taxifolin could act as a potential therapeutic agent for CAA.

## CAA Inducing Cerebrovascular Disease

**FIGURE 1:**
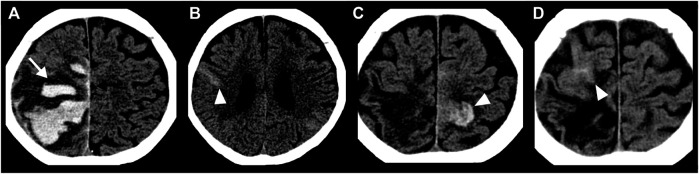
Head CT images of an 84-year-old woman with CAA showing repeated non-traumatic intracranial hemorrhage over four months. **(A)** ICH presenting finger-like projections (white arrow) with SAH extension (black arrow). **(B)** Acute convexity SAH (arrowhead) at two months later than **A**. **(C)** Acute convexity SAH (arrowhead) at three months later than **A**. **(D)** Acute convexity SAH (arrowhead) at four months later than **A**.

CAA induces smooth muscle cell degeneration, vessel wall thickening, luminal narrowing and concentric wall splitting, resulting in varying degrees of intracerebral hemorrhage (ICH) (Love et al., 2014). Lobar, but not deep, ICH is associated with CAA (Saito et al., 2020a). Finger-like projections and subarachnoid hemorrhage extension of lobar ICH, together with the ApoE4 genotype, are reliable diagnostic markers for CAA (Rodrigues et al., 2018; Renard et al., 2019) (Figure 1A). Cerebral microbleeds (CMBs) are commonly observed in patients with CAA. Strictly lobar CMBs are highly specific for CAA, while CMBs in deep brain may indicate hypertensive arteriopathy (Greenberg and Charidimou, 2018; Jung et al., 2020). The estimated annual incidence of CAA-related ICH is 5.3 per 100,000 people in the United Kingdom and 5.8 per 100,000 people in Japan; however, the incidence of ICH not resulting from CAA, and mainly associated with hypertensive arteriopathy, is 2.5-fold higher in Japan than the United Kingdom (Yakushiji et al., 2020). Early diagnosis of CAA is clinically important for guiding prognosis and treatment decisions. A recent prospective study, with a median follow-up time of 2.5 years, showed progression to dementia in more than 25% of patients with CAA-related ICH, even if no dementia had presented after the acute phase of ICH (Xiong et al., 2019). ICH recurrence was more frequent in patients with CAA than other potential causes (Pasi et al., 2018).

CAA is likely clinically underdiagnosed, due to the various clinical presentations outside of lobar ICH (Sakai et al., 2019; Fakan et al., 2020). Subarachnoid hemorrhage (SAH), resulting from bleeding into the subarachnoid space, known as “convexity SAH” in the acute phase (Figures 1B–D) and “superficial siderosis” in the chronic phase, can be induced by CAA (Saito et al., 2020a). Most CAA-induced bleeding into the subarachnoid space is limited without the involvement of the adjoining brain parenchyma (Kumar et al., 2010). Many convexity SAH are asymptomatic, though the risk of future intracranial hemorrhage and death of patients with CAA-convexity SAH is very high (Calviere et al., 2019; Saito et al., 2020a).

CAA also induces ischemic strokes consisting of both macro and microinfarcts (Saito et al., 2015; Saito and Ihara, 2016). Cerebral microinfarcts were originally defined as infarcts only visible by microscopy (Okamoto et al., 2012). However, technological advances in imaging modalities, such as ultra-high-field MRI, have enabled cerebral microinfarct observation (Ishikawa et al., 2020; Ter Telgte et al., 2020). AD and CAA patients frequently possess cortical cerebral microinfarcts near Aβ-laden vessels (Okamoto et al., 2009; Okamoto et al., 2012). Cerebral microinfarcts were replicated in CAA model mice following chronic cerebral hypoperfusion by bilateral common carotid artery stenosis (Okamoto et al., 2012). Impaired vasodilation due to vascular Aβ accumulation may contribute to cerebral microinfarct pathogenesis. AD and CAA patients have numerous, sometimes exceeding 1,000 (Westover et al., 2013), cerebral microinfarcts (van Rooden et al., 2014), which are likely to contribute to cognitive impairment (Saito et al., 2015).

## CAA as a Contributor to Neurodegenerative Disorder

CAA plays a pivotal role in the pathogenesis of dementia and is independently associated with cognitive decline (Boyle et al., 2015; Banerjee et al., 2018). Since there is little evidence for overproduction, the failure of clearance of Aβ peptides is a likely key factor in the pathological development of AD and CAA (Mawuenyega et al., 2010; Iturria-Medina et al., 2014). There is therefore increasing interest in developing agents that promote the safe elimination of Aβ from the brains of aged people (Saito and Ihara, 2014). The necessity of promoting Aβ clearance has been demonstrated in clinical trials using Aβ immunization. In AN-1792-vaccinated AD patients, the number and extent of parenchymal Aβ plaques diminished, while cerebrovascular Aβ accumulation and CAA increased (Nicoll et al., 2003; Patton et al., 2006). This finding was also observed in patients treated with solanezumab, a monoclonal anti-Aβ antibody (Roher et al., 2016). Antibody-solubilized Aβ appears to be removed from the cortex and re-deposited in the walls of the cerebral blood vessels via intramural periarterial clearance pathways (Carare et al., 2020).

**FIGURE 2:**
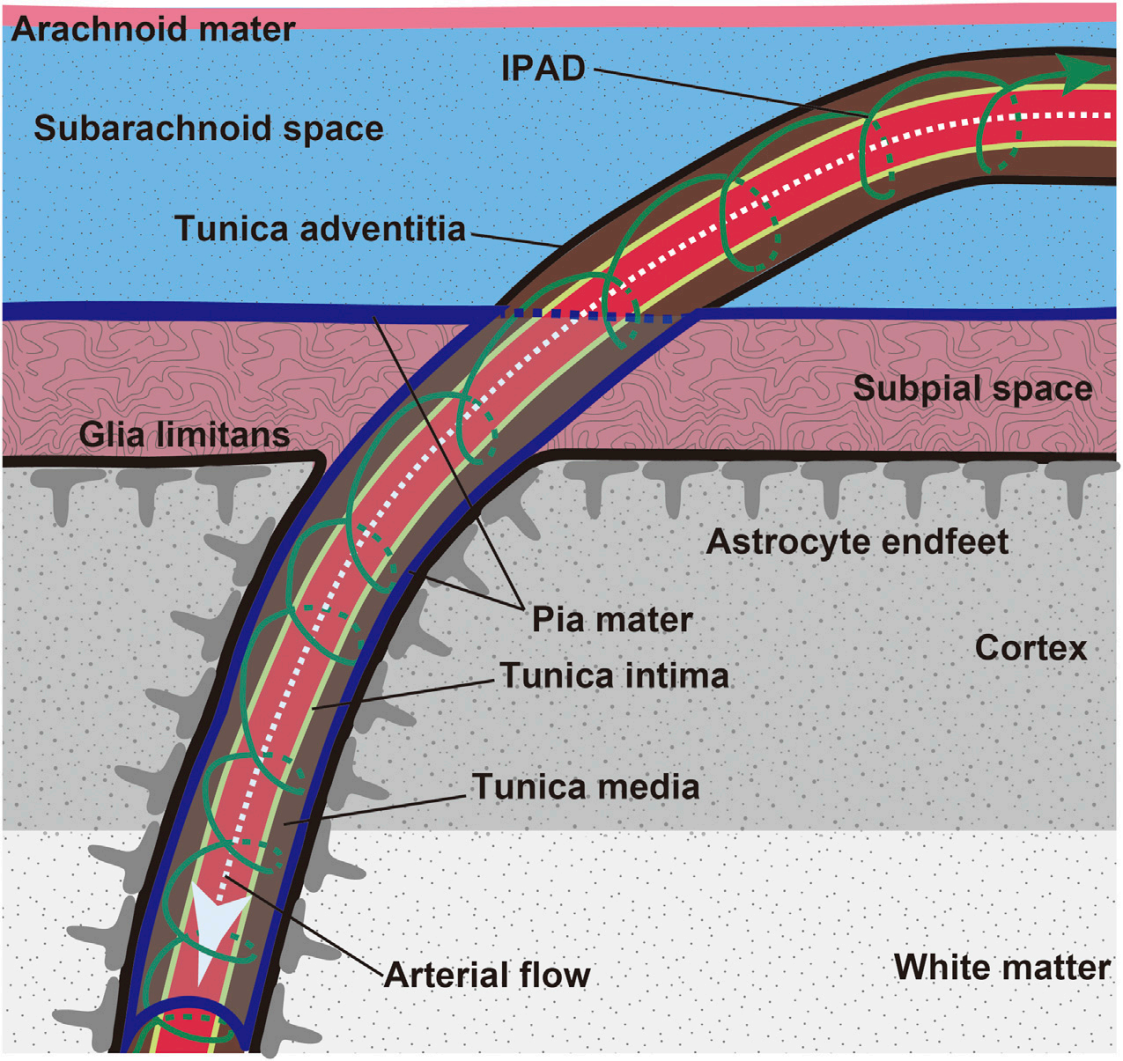
Scheme of leptomeninges and penetration of leptomeningeal artery into the brain parenchyma. Brain waste is cleared through the IPAD route (green arrow). Aβ is preferentially deposited in the tunica media of the leptomeningeal and cortical arteries in CAA.

Intramural periarterial drainage (IPAD), is a mechanism for the drainage of fluid and solutes from the brain along the walls of cerebral arteries (Tarasoff-Conway et al., 2015; Saito et al., 2019) (Figure 2). The central nervous system is devoid of lymph vessels. Instead, interstitial fluid and solutes within the extracellular matrix, including soluble Aβ, enter the IPAD pathways within the basement membranes of capillaries and continue to the basement membranes surrounding smooth muscle cells (SMCs) of the intracerebral and leptomeningeal arteries (Carare et al., 2020), which lead to the cervical lymph nodes (Piotrowska et al., 2020). This process has been examined in detail by several imaging methods including electron (Morris et al., 2016), confocal (Carare et al., 2008; MacGregor Sharp et al., 2020) and two-photon (Arbel-Ornath et al., 2013; Kim et al., 2020), microscopy. IPAD flow rapidly moves toward the leptomeningeal arteries where the deposition of Aβ is prominent in CAA (Keable et al., 2016). Aβ levels in the cerebrospinal fluid are decreased in CAA (Verbeek et al., 2009; van Etten et al., 2017), suggesting that Aβ transport is impeded in the IPAD pathways.

Transcytosis is another vascular-mediated Aβ clearance system closely associated with AD and CAA. The brain parenchyma is separated from capillary lumen by the blood brain barrier (BBB), which prevents passive exchange between the brain and blood, allowing controlled carrier-mediated bidirectional transport of nutrients and waste products (Sweeney et al., 2018; Sweeney et al., 2019). Several molecules, such as low-density lipoprotein receptor related protein-1 (LRP-1), are thought to be involved in Aβ efflux from brain to blood (Shibata et al., 2000; Deane et al., 2004). Aβ binds to LRP-1 at the abluminal side of the vascular endothelium, either as a free peptide or bound to ApoE2 and ApoE3. Aβ-ApoE2 and Aβ-ApoE3 complexes are rapidly cleared across the BBB into the blood, while Aβ bound to ApoE4 interacts poorly with LRP-1 and is less efficiently removed from brain (Deane et al., 2008). Aβ deposition is frequently found in the cerebral capillaries in subjects possessing the ApoE4 allele (Thal et al., 2008).

CAA is not merely a consequence of impaired IPAD or transcytosis but also an important contributor to these processes (Kim et al., 2020; Rosas-Hernandez et al., 2020). CAA damages arterial structure and function, leading to worsening of cerebrovascular function and cognition. Therefore, early intervention strategies against CAA could be key to preventing progression of AD.

### Challenges in Developing Novel Therapies for CAA

Development of novel treatments for CAA has proved challenging, with no pharmaceutical agents currently available (Smith and Markus, 2020). While more than 100 trials are in progress for AD (Cummings et al., 2020), to our knowledge, there are no ongoing clinical trials for agents targeting CAA (The U.S. National Institutes of Health, 2020), though a clinical trial of minocycline, a tetracycline derivative with anti-inflammatory properties is being planned in the Netherlands. Previous clinical trials on agents targeting CAA have reported mixed findings. Tramiprosate (3-amino-1-propanesulfonic acid), a low-molecular-weight ionic compound with preferential binding to soluble form of Aβ, has been shown to effectively block the deposition and facilitate the clearance of Aβ from the brains of transgenic mice expressing a double mutant (K670N/M671L and V717F) human *APP* gene (Gervais et al., 2007) but does not bind to insoluble fibrillar Aβ (Gervais et al., 2007). However, a phase-II trial of tramiprosate demonstrated no beneficial effects on CMBs despite causing no major safety issues (Greenberg et al., 2006; Gauthier et al., 2009; Aisen et al., 2011; Smith and Markus, 2020). In another trial, the anti-Aβ-monoclonal antibody, ponezumab was investigated in patients with CAA (Leurent et al., 2019). Ponezumab was well tolerated and plasma levels of Aβ_40_ were increased in the ponezumab-treated group, suggesting effective removal from the brain. However, ponezumab did not improve visual task-related functional MRI activation, a marker for cerebrovascular reactivity (Leurent et al., 2019).

### Taxifolin for CAA

Taxifolin is emerging as a viable safe therapeutic agent for the prevention and treatment of CAA. Taxifolin, also known as dihydroquercetin, is a bioactive flavanonol commonly found in grapes, citrus fruits, onions, green tea, olive oil, wine and several herbs such as milk thistle, French maritime bark, Douglas fir bark, and Smilacis Glabrae Rhizoma (Yang et al., 2016). Taxifolin is also widely used as a food additive and can be found in health supplement products including silymarin (Yang et al., 2016). Taxifolin has received increasing attention as a potential treatment for various diseases such as cancer, cardiovascular diseases, viral hepatitis, dyslipidemia and neurodegenerative disorders (Weidmann, 2012). It exhibits various pharmacological effects (Sunil and Xu, 2019), including anti-oxidant (Guo et al., 2015), advanced glycation end product suppressing (Harris et al., 2011), and mitochondrial protecting (Haraguchi et al., 1996) properties. Inhibition of Aβ fibril formation by Taxifolin has been demonstrated by using transmission electron microscopy imaging (Sato et al., 2013a; Sato et al., 2013b; Saito et al., 2017). Thioflavin T fluorescence assays have also shown that aggregated Aβ fibrils can be disaggregated by Taxifolin (Sato et al., 2013a), seemingly due to its chemical structure properties. Taxifolin is oxidized to form *o*-quinone on its B-ring. Since Lys16 and Lys28 are involved in the formation of β-sheets of Aβ, oxidized Taxifolin prevents the aggregation of Aβ as it covalently binds to Aβ at Lys16 and Lys28 residues (Sato et al., 2013b; Tanaka et al., 2019).

Aβ disassembly by Taxifolin treatment was confirmed *in vivo*. We administered Taxifolin or vehicle to a mouse model of CAA expressing the human *APP* gene with Swedish/Dutch/Iowa triple mutations (Saito et al., 2017). Filter trap assays showed a significant decrease in the concentration of Aβ oligomer in the soluble fraction of brain of Taxifolin-treated mice (Saito et al., 2017). However, the amount of total Aβ in the soluble fraction was similar between the Taxifolin-treated and vehicle-treated CAA mice, suggesting Taxifolin prevented the formation of Aβ oligomers from monomers (Saito et al., 2017). Furthermore, Taxifolin treatment prevented spatial memory deficits induced by injection of oligomeric Aβ into the hippocampus of wild-type mice (Wang et al., 2018). Decreased levels of Aβ oligomers by Taxifolin treatment were seen even in advanced stages of CAA (Saito et al., 2017). Higher blood Aβ levels were found in Taxifolin-treated CAA mice, suggesting facilitation of Aβ clearance from brain to blood. Taxifolin also fully restored both cerebrovascular reactivity and spatial reference memory in CAA mice (Saito et al., 2017). Higher expression of triggering receptor expressed on myeloid cell 2 (TREM2) is associated with the inflammation in the brain (Tanaka et al., 2020). We reported that Taxifolin suppressed inflammation, alleviating the accumulation of TREM2-expressing cells in the brains of CAA model mice (Inoue et al., 2019). Furthermore, Taxifolin suppressed glutamate levels and oxidative tissue damage, resulting in the amelioration of apoptotic cell death. In short, Taxifolin exhibits pleiotropic neuroprotective effects against CAA (Inoue et al., 2019).

## Future Perspective

In light of the promising preclinical data outlined in this review, we are currently preparing a clinical trial of Taxifolin in CAA patients. Nevertheless, several caveats on the use of this exciting potential treatment option should be addressed. Firstly, preclinical studies have not demonstrated that Taxifolin mitigates or prevents ICH, suggesting this may represent an inappropriate efficacy outcome in a clinical trial. Considering that Taxifolin restores cerebrovascular reactivity in mice (Saito et al., 2017), improvement of the cerebrovascular reserve capacity may be a more suitable in evaluating efficacy in CAA patients; indeed, impaired vascular reactivity is an early manifestation of CAA (van Opstal et al., 2017). However, the use of a surrogate, instead of a clinical, endpoint as a primary outcome of the efficacy of a drug in a clinical trial of a common disease such as CAA remains controversial (Broich et al., 2011). Secondly, the optimal dose and usage of Taxifolin in humans should be assessed. We reported the inhibition of Aβ oligomer formation in mice using a high dose of Taxifolin (Saito et al., 2017). In our experiments, 3% Taxifolin was administered orally to mice weighing approximately 30 g and consuming 3–5 g chow a day. It is still unknown whether smaller doses of Taxifolin initiate Aβ disassembly *in vivo*. Daily doses of 100 mg per day of Taxifolin are frequently administered as a health supplement product. However, whether high doses of Taxifolin are safe and tolerated in humans has yet been established and the elimination half-life period of Taxifolin is short at less than 1 h (Saito et al., 2017). Thirdly, as many as 191 metabolites of Taxifolin were reported in rats (Yang et al., 2016). Given that some of the metabolites could exhibit anti-CAA effects as well as Taxifolin, individual differences in the metabolism of Taxifolin may affect the response on Aβ disassembly in each patient, meaning the safety of such derivatives should be also evaluated. Finally, the identification of predictive indicators of favorable response of Taxifolin on CAA should be prioritized. Heterogeneity and multimorbidity are common in the elderly (Barnett et al., 2012), meaning the pharmacokinetic and pharmacodynamic effects of Taxifolin may vary in different individuals. However, the grouping of the patients based on the results of predictive indicators may facilitate more targeted, stratified or precision medicine treatments (Hampel et al., 2018).

## Conclusion

Although numerous agents derived from natural plants now play pivotal roles in the prevention and treatment of various diseases, the importance of medicinal plant research may be underestimated in the field of AD and CAA. The beneficial effects demonstrated in preclinical studies suggest more promise for the clinical use of Taxifolin than other drug candidates for CAA. Future basic and clinical studies of this commonly used bioactive flavonoid could open new avenues for preemptive medicine for AD and CAA.
